# Where to Begin: Assessing Caregiver Readiness

**DOI:** 10.1093/geroni/igaa057.2414

**Published:** 2020-12-16

**Authors:** Katherine Marx, Laura Gitlin

**Affiliations:** 1 Johns Hopkins University, Baltimore, Maryland, United States; 2 Drexel University, Philadelphia, Pennsylvania, United States

## Abstract

There have been many interventions targeting family caregivers for people living with dementia (PLwD) that have been found to have efficacy and effectiveness. However, very few of these interventions have been widely adopted. One reason may be that clinicians and other professionals working with caregivers are unsure of what intervention to use. One measure that may help with where to begin is Caregiver Readiness. This presentation will provide case studies on the use of the Caregiver Readiness Scale in one intervention, the Tailored Activity Program (TAP). We will highlight three cases that have low, moderate and high readiness scores and how the TAP interventionists use the readiness scores to tailor the intervention to match the caregiver’s need. Knowing a caregiver’s readiness to receive an intervention may help clinicians and providers identify where to being in helping the caregiver.

